# Development of a Caregivers’ Support Platform (Connected Health Sustaining Home Stay in Dementia): Protocol for a Longitudinal Observational Mixed Methods Study

**DOI:** 10.2196/13280

**Published:** 2019-08-28

**Authors:** Estefania Guisado-Fernandez, Brian Caulfield, Paula Alexandra Silva, Laura Mackey, David Singleton, Daniel Leahy, Sébastien Dossot, Dermot Power, Diarmuid O'Shea, Catherine Blake

**Affiliations:** 1 Insight Centre for Data Analytics School of Public Health, Physiotherapy and Sports Science University College Dublin Dublin Ireland; 2 DigiMedia Research Center Universidade de Aveiro Aveiro Portugal; 3 School of Public Health, Physiotherapy and Sports Science University College Dublin Dublin Ireland; 4 Applied Research for Connected Health University College Dublin Dublin Ireland; 5 Medicine for the Older Person Mater University Hospital Dublin Ireland; 6 Department of Geriatric Medicine St. Vincent's University Hospital Dublin Ireland

**Keywords:** connected health, dementia, caregivers, home care, home monitoring

## Abstract

**Background:**

Dementia disease is a chronic condition that leads a person with dementia (PwD) into a state of progressive deterioration and a greater dependence in performing their activities of daily living (ADL). It is believed nowadays that PwDs and their informal caregivers can have a better life when provided with the appropriate services and support. Connected Health (CH) is a new technology-enabled model of chronic care delivery where the stakeholders are connected through a health portal, ensuring continuity and efficient flow of information. CH has demonstrated promising results regarding supporting informal home care and *Aging in Place*, and it has been increasingly considered by researchers and health care providers as a method for dementia home care management.

**Objective:**

This study aims to describe the development and implementation protocol of a CH platform system to support informal caregivers of PwDs at home.

**Methods:**

This is a longitudinal observational mixed methods study where quantitative and qualitative data will be combined for determining the utility of the CH platform for dementia home care. Dyads, consisting of a PwD and their informal caregiver living in the community, will be divided into 2 groups: the intervention group, which will receive the CH technology package at home, and the usual care group, which will not have any CH technology at all. Dyads will be followed up for 12 months during which they will continue with their traditional care plan, but in addition, the intervention group will receive the CH package for their use at home during 6 months (months 3 to 9 of the yearly follow-up). Further comprehensive assessments related to the caregiver’s and PwD’s emotional and physical well-being will be performed at the initial assessment and at 3, 6, 9, and 12 months using international and standardized validated questionnaires and semistructured individual interviews.

**Results:**

This 3-year funded study (2016-2019) is currently in its implementation phase and is expected to finish by December 2019. We believe that CH can potentially change the PwD current care model, facilitating a proactive and preventive model, utilizing self-management–based strategies, and enhancing caregivers’ involvement in the management of health care at home for PwDs.

**Conclusions:**

We foresee that our CH platform will provide knowledge and promote autonomy for the caregivers, which may empower them into greater control of the care for PwDs, and with it, improve the quality of life and well-being for the person they are caring for and for themselves through a physical and cognitive decline predictive model. We also believe that facilitating information sharing between all the PwDs’ care stakeholders may enable a stronger relationship between them, facilitate a more coordinated care plan, and increase the feelings of empowerment in the informal caregivers.

**International Registered Report Identifier (IRRID):**

DERR1-10.2196/13280

## Introduction

### Background

Dementia is a neurodegenerative chronic condition that is frequently described as a clinical syndrome characterized by cognitive and functional decline accompanied by changes in a person’s behavior and personality, which interferes with social or occupational functioning [1]. As the World Health Organization (WHO) reports, an estimated 47 million people worldwide were affected in 2015, and these numbers are expected to increase to 75 million by 2030 and 132 million by 2050 [2]. This was one of the main reasons for the WHO to designate dementia as a public health priority in 2012 [3], launching a public health dementia plan in 2017 [2]. Dementia is considered one of the most disabling chronic diseases that leads the person with dementia (PwD) into a state of progressive deterioration and a greater dependence in performing their activities of daily living (ADLs).

A dementia diagnosis also has a significant impact on the PwD’s family who often bear the responsibility of caring for them as their condition progresses. Family members, usually a spouse or a child, are often referred to as *informal caregivers*, as they provide unpaid and continuous assistance contrary to formal caregivers who are paid for their professional services [4]. Informal caregiving can help to maintain the PwD at home, avoiding institutionalization and providing the *Aging in Place* new model of care, which consists of helping older people to remain living at home for as long as possible; avoiding nursing home placement; and contributing to an increase in well-being, independence, social participation, and healthy aging [5]. However, along with the well-being and economic improvement that *Aging in Place* can offer, previous research has also shown that the majority of people older than 75 years prefer living independently at home for as long as possible [6].

Although it is recognized that dementia can bring many challenges for the PwDs and their caregivers, it is also believed that nowadays they can have a better life when provided with the appropriate diagnosis and the proper services and support [7]. Connected Health (CH) is a new technology-enabled model of chronic care delivery where the stakeholders are *connected* through a health portal, ensuring continuity and efficient flow of information [8]. CH has demonstrated promising results regarding supporting informal home care and *Aging in Place* [9,10]. It represents an opportunity for passive and unobtrusive home monitoring, and increasingly, it is being considered by researchers and health care providers to be an enabler for dementia home-based health and wellness measurement and monitoring using mobile devices for storage, update, and transmission of patient data. The data captured from everyday life in PwDs can be of great value when put in the hands of the appropriate stakeholders, such as the health care professionals and the informal caregivers, as it can help to track the progress of PwDs and act as a predictor of any cognitive or physical decline [11]. Furthermore, reductions in PwD’s caregiver burden, stress, depression, and anxiety as well as improved self-efficacy and confidence regarding caregiving skills have also been found; hence, it is believed that these results can be extrapolated to PwDs and that the implementation of these technologies may then improve the quality of life for both the PwD and their caregiver [12].

In terms of technology-driven interventions for dementia care, recent research shows that CH can improve various aspects of informal caregiving, such as confidence, depression, and self-efficacy, through the provision of multiple components personalized to each individual. In addition, informal caregivers can benefit from the interaction with other peers or health care professionals [13]. Focusing on the informal caregiver support provided, we found multiple types of technologies in the literature that can be categorized under the umbrella term of CH, including assisted living technology or ambient assisted living technologies, information and communication technology, smart homes, telehealth, telemonitoring, mobile health, and electronic health. Their purpose is to support the informal dementia caregiver in the physical, emotional, and social spheres of their role. As a result, the main outcome of interest of the studies where these technologies are used was an improvement in caregiver’s mental and physical well-being, comparing before and after deployment, as measured by caregiver’s stress, anxiety, depression, burden, and quality of life [14]. A systematic review conducted by Godwin et al [14] on technology-driven interventions for caregivers of PwDs found that these interventions showed positive results in the potential reduction of the caregivers’ burden, improvement in their mental health, increase in their caregiving skills, and increase in competence as a direct benefit from online education, internet-based support groups, computer-mediated interactive voice response systems, and online skill building [14]. This is in line with the current trends of digital interventions for facilitating patients’ and caregivers’ empowerment [15-17]. Another reason for this growing interest for CH in supporting dementia home management is that it can offer a cost-effective alternative to face-to-face care, and it has been progressively integrated into hospitals, physician’s offices, patient homes, and other settings. At the same time, it allows access to personalized health education and support for self-management [18].

A good example found in the literature using a combination of patient home monitoring and informal caregiver support is the ALADDIN (Assisted living of Dementia elDerly INdividuals and their carers) project, which was conducted by Torkamani et al in 2014 [12]. ALADDIN is a computerized platform designed to offer support and information to the carer. It also manages and communicates information related to the PwD and their carers from their home to the clinicians, facilitating distant monitoring. It was tested in a multisite randomized controlled pilot study with 30 community-living informal caregivers of PwDs. The intervention and control groups were assessed at baseline and at 3 and 6 months in terms of burden, depression, and quality of life for the caregiver and in terms of cognitive and disease stage, functional disability, comorbidities, and quality of life for the PwD. The authors reported a significant improvement in the quality of life of the carers in the platform group, with some reduction in carer burden and distress, and that the platform was useful in monitoring the patients and facilitating contact with other professionals. In addition, carers and clinicians rated the access to and use of the ALADDIN platform positively. The success of studies such as this supports further testing of the utility and value of CH in other dementia cohorts and for longer periods and investigating the impact that it can have in the care of PwDs.

### Study Aim

This paper describes the development and implementation protocol of a CH platform system to support informal caregivers of PwDs at home. We believe that a better understanding and more involvement in dementia home care can improve the informal caregivers’ caring experience along with their quality of life and well-being. We want to explore if CH can offer additional support to traditional home care, promoting PwD and caregiver well-being and reducing the amount of burden and stress experienced by the caregiver, which may be translated into better care coping. In addition, we want to gain an understanding of the potential barriers and enablers to adoption by PwD and their informal caregivers of a CH platform to uncover how to change potential negative attitudes toward technology (behavioral change).

## Methods

### Study Design

This paper reports the protocol for a project based on a pilot study conducted by Applied Research in Connected Health, University College Dublin (UCD), between March and June 2014, where technology was deployed to 28 PwD and caregiver dyads for 6 weeks. The pilot project implemented a CH model prototype into dementia home care to investigate its feasibility and acceptability among PwDs and their informal caregivers. The following home-monitoring devices were deployed in the participants’ homes for monitoring the PwD (Multimedia Appendix 1): an Omron blood pressure (BP) monitor (Omron Healthcare, Kyoto, Japan), a Withings Pulse activity tracker (Withings consumer electronics, Paris, France), a Withings scale (Withings consumer electronics, Paris, France), a ResMed Sleep Minder (Biancamed Ltd, Dublin, Ireland), and an Android tablet with the online health platform where all the data collected from the monitoring devices were uploaded and made available for the informal caregivers and the PwD’s health care professionals. Furthermore, the health platform included a section with dementia disease information videos, an online diary section, and a questionnaires section where informal caregivers could periodically fill out some questionnaires regarding the PwD’s ADLs. During the course of the development, the researchers communicated with PwDs and caregivers via video conferencing using the Android tablet provided. Further comprehensive assessments were conducted before and after the CH devices deployment for measuring the informal caregivers’ and PwDs’ quality of life, stress, and depression using scientifically validated questionnaires. Participants were recruited from 2 university hospitals in Dublin that work in collaboration with UCD. Results from its implementation and evaluation in terms of usability and user experience were used to develop the longitudinal project study design that is explained next.

The knowledge acquired from the literature review and the pilot study was applied in the design and development of *Connected HEalth Sustaining home Stay in Dementia* (CHESS), a longitudinal observational mixed methods study where quantitative data will be combined with qualitative data for determining the utility of a CH platform for dementia home care. For this purpose, 3 main objectives have been defined: (1) project feasibility—to evaluate the effectiveness of the CH platform in supporting caregivers of PwDs compared with usual care and caregivers’ ability to cope; (2) impact of CH in dementia home care—in terms of the PwD and their informal caregivers’ physical and mental health, quality of life, and well-being; and (3) users’ feedback—to determine the CH platform’s usability and user experience from the caregivers’ perspectives.

### Participants: Inclusion and Exclusion Criteria

Dyads consisting of a PwD and their informal caregiver living in the community are eligible for this study. For inclusion, the PwD must have a confirmed diagnosis of dementia by a health care professional, have a Mini-Mental State Examination (MMSE) score of 10 or above, have a live-in or dedicated informal caregiver (living with the PwD or spending with them at least 5 days a week, with no minimum hours per day requirement), be 65 years or older, be fluent in English (verbally and written), have a life expectancy of more than 6 months with no hospital admissions in the 3 months before enrolment, have adequate hearing and vision, and not be actively involved in another research study.

Caregivers must be live-in or dedicated informal caregivers, be fluent in English (verbally and written), have adequate hearing and vision, have a life expectancy of more than 6 months, and not be involved in another research study.

In case of not having an internet connection at home, it will be provided to the participants. Therefore, this has not been considered as a selection criterion.

### Ethical Approval, Participants’ Recruitment, and Seeking Consent

Ethical approval from the participating hospitals where recruitment will take place has been granted. Informed consent will be obtained on an individual basis in accordance with legal and ethical guidelines, following careful explanation and provision of an informed consent form for the PwD and a separate informed consent form for the caregiver, detailing their level of involvement. This provided informed consent and cognitive status will be reviewed before each quarterly assessment, giving the PwD and their informal caregiver the right to withdraw at any time. In case the cognitive status decreases under the minimum inclusion score of 10 points or their health declines significantly, they will be asked to abandon the study.

### Intervention Description

This will be a longitudinal cohort study with 2 groups: the *intervention group*, which will receive the CH technology package at home, and the *usual care* group, which will not have any CH technology package at all. Selection to the CH or usual care group will be made by the PwD and their informal caregiver; thus, we will have adopter (intervention) and nonadopter (usual care) groups. The volunteer PwD-caregiver dyads will freely choose in which group they would like to participate as we acknowledge that this is a vulnerable population at an advanced age who might not be technology educated or might not feel comfortable with technology.

The CH technology package (Multimedia Appendix 2) consists of an online health platform designed for the informal caregiver that runs in a tablet computer (Samsung Galaxy Tab A 10.1, 2016) connected to a range of PwD monitoring devices for home use, including a BP monitor (Omron M6), an electronic weighing scale (Withings), and an activity and sleeping tracker (Withings Go). The online platform includes 4 sections (Multimedia Appendix 3). The first (Multimedia Appendix 4) consists of an information and resource section that provides access to website links and videos from Irish dementia experts with reputable information regarding dementia management and helpful advice about daily home care. The second section (Multimedia Appendix 5) provides daily and weekly questionnaires that gather health-related information about the PwD, that is, mood, nutrition, activity, bowel movements, and medication compliance, which are completed by the caregiver and viewed by the health care professionals involved in the PwD’s care. In addition, questionnaires for the informal caregivers are included to assess their own health and well-being, that is, mood, energy levels, sleeping quality, and anxiety levels. This will be used for research purposes only and not viewable for health care professionals. The third section (Multimedia Appendix 6) includes a journal to be used as an online diary where the informal caregivers can maintain recordings of events, and it also contains summary reports of changes in the PwD care plan provided by the health care professionals from previous consultations. The fourth section (Multimedia Appendix 7) offers a dashboard for the caregivers and health care professionals to view the PwD activity levels, sleep patterns, BP, and weight, recorded by the monitoring devices provided along with the health platform. During the deployment, the informal caregiver has access to the health platform at any time using the tablet computer. They will have to measure the PwD’s BP once a week and input the results into the *measurements* section. Safety range values of each individual BP will be set up in the health platform during the first assessment, so this measurement will act as their reference BP value. If BP measurements fall outside these parameters (2 points above or below the first assessment values), a message will pop up on the tablet screen advising the informal caregivers to check how the PwD is feeling at that moment and to take another BP measurement in 30 min. Following this, if the value measured is still outside the normal limits and the PwD is feeling unwell, they will be advised to contact their general practitioner. Weight has to be measured by the informal caregiver once a month and will be automatically uploaded from the scale to the health platform via Wi-Fi. Daily activity and sleeping, tracked from the activity wrist tracker, is automatically uploaded via Bluetooth into the health platform. In addition, the informal caregivers will be prompted through messages under each section name to complete the daily and weekly questionnaires on the platform, to take the weight and BP, and to have a look at the information section, which will be updated with new resources regularly.

The encrypted online platform will securely connect all the key stakeholders involved in the PwD’s care (ie, informal caregiver, general practitioner, public health nurse, and hospital geriatric services). As mentioned above, the generated data will be presented in the platform and made available for the informal caregivers and the health care professionals as an objective measure of the patient’s health status.

### Outcomes Measurements

A multidimensional profile of the well-being of caregivers and PwDs will be tracked longitudinally over the duration of the study, with some brief measures recorded daily (questionnaires and PwD’s activity and sleeping), another weekly (BP) or monthly (weight), and others at quarterly intervals (assessments questionnaires). These measures will be contrasted between the intervention and usual care groups at discrete time points and over time. The daily and weekly monitoring will consist of simple Likert-type questions about the PwD and caregiver (completed by the caregiver) along with the physical activity of the PwD measured in steps and sleep duration.

More detailed outcomes will be compared and evaluated through international validated questionnaires administered by the researcher and that are commonly used in the clinic to measure well-being. Choosing the appropriate questionnaires and the right time for completing them was decided after a concise literature review that also required meetings and advice from experts in dementia and considerations of the demands on the caregiver and PwD. Our outcome domains can be divided into 2 groups that are described below (Multimedia Appendix 8). In addition, qualitative interviews will take place at months 3 and 9 of the year of deployment to analyze caregivers’ satisfaction with the CH platform along with the usability and user experience.

#### Person With Dementia–Related Outcomes

The cognitive status level in the PwD will be measured using the *Mini-Mental State Examination* (MMSE) questionnaire. It is composed of 11 questions that cover 5 areas of cognitive function: orientation, registration, attention and calculation, recall, and language. The maximum score is 30, with a score of 23 or less being indicative of cognitive impairment. This is a quick and easy tool to administer directly to the PwD and is very useful when having to conduct it repetitively [19,20].

Behavioral and neuropsychiatric symptoms will be assessed with the short version of the *Neuropsychiatric Inventory Questionnaire* (NPI-Q). It is a way of providing a brief assessment of the PwD’s neuropsychiatric symptoms, which has been seen to be sensitive and reliable to capture changes in time related to delusions, hallucinations, agitation or aggression, depression or dysphoria, anxiety, elation or euphoria, apathy or indifference, disinhibition, irritability or lability, aberrant motor behavior, sleep and night-time behavior disorders, and appetite and eating disorders. It is based on responses of the caregivers who must also rate these symptoms in terms of frequency (how frequent those symptoms occur), severity (how severe and how much it affects the PwD), and distress (how distressing is it for them as caregivers and how much it may affect them). The NPI-Q does not have a cutoff score and, therefore, does not have a numeric scoring. Each domain is scored for frequency, severity, and associated caregiver distress, and the interviewer has to analyze them in those given terms [21-23].

Quality of life will be measured with the *Dementia Quality of Life* (DEMQOL) questionnaire, to gain an insight into their health-related quality of life. This is designed to work across dementia subtypes and care arrangements and is suitable for all the stages of the disease. It is composed of 2 questionnaires: (1) DEMQOL, a 28-item questionnaire answered by the PwD (self-reported quality of life) and (2) DEMQOL-Proxy, a 31-item questionnaire answered by the caregiver (PwDs’ caregiver- reported quality of life). Scored items are summed to produce a total score, with higher scores meaning a better health-related quality of life [24,25].

Anxiety and depression levels will be examined using the *Hospital Anxiety and Depression Scale* (HADS), a simple and brief self-reported questionnaire. A total summary score classifies the respondent into 3 groups, depending on their levels of depression and anxiety, into normal, borderline case, or abnormal. This questionnaire does not provide a diagnosis as it was created for screening purposes only [26,27].

PwD functional levels will be assessed with the *Disability Assessment for Dementia* questionnaire, designed for community-based individuals with Alzheimer dementia type, but it has been used in other types of dementia research more recently. It is a tool used by health care professionals to investigate the PwD levels of dependency and to guide the provision of tailored interventions for PwD. In addition, as a research tool, it can be used to describe the functional characteristics of the PwD and the progression of the disease. A total score is converted out of 100, with the result of a percentage that gives an understanding of the PwD global function in ADLs. Higher scores mean less disability in conducting ADLs, with lower scores indicating more dysfunction and more dependency on the caregiver [28].

PwD frailty will be examined with the *Survey of Health, Ageing, and Retirement in Europe Frailty Instrument (SHARE-FI)*. It is a 5-item validated scale created by Romero-Ortuno et al [29,30], which predicts frailty and mortality in primary care similarly to a frailty index based on a comprehensive geriatric assessment. It is quick and easy to administer as it only requires 5 simple measurements to be entered in an open-access online calculator, which we have incorporated into our health platform.

PwDs’ risk of falls; gait, and mobility assessment will be conducted with *Kinesis Quantitative Timed Up and Go (QTUG*). It is an online app tool for the QTUG test, validated through research by the company *Kinesis* that measures different phases of the *Timed Up and Go* test [31]; time in turning; and reflects overall locomotor task ability, variability, and stability. QTUG can identify a large range of temporal gait parameters. Using an internal algorithm integrates these with clinical falls risk indicators to calculate a falls risk score [32,33].

#### Caregiver-Related Outcomes

Quality of life in the caregiver will be measured using the EuroQol-5 dimention-5 level instrument (*EQ-5D-5L*) [34]. It is a standardized measure of health status, creating a simple descriptive profile and a single index value that can be used for the clinical and economic evaluation of health status and health care. It consists of 2 parts. The first part is the descriptive system that comprises 5 dimensions, mobility, self-care, usual activities, pain/discomfort, and anxiety/depression, each of these having 5 possible response options: no problems, slight problems, moderate problems, severe problems, and extreme problems. The interviewee must choose 1 answer that best represents their health state. The second part is the visual analogue scale (EuroQol group Visual Analogue Scale, EQ-VAS), which is a 20-cm vertical scale scoring from 0 to 100, with endpoints marked with “the best health you can imagine” for the 100 score and “the worst health you can imagine” for the 0 score. The interviewee has to mark out a point in the scale, providing a score representing their self-rated score. From the first part, an individual health state is defined for each respondent combining 1 level from each of the 5 dimensions, giving a personalized digit code for their health status. The second part, the EQ-VAS, provides a quantitative measure of the interviewed health status.

Anxiety and depression levels will be assessed using the same tool as applied for the PwD, the HADS, which is described above.

The burden of care and ability to cope will be estimated using the *Zarit Burden Interview questionnaire*. It is composed of 22 questions about the impact of the PwD’s disabilities in the caregiver’s life and has been designed to reveal the stress experienced by the caregiver. For each item, the caregivers must indicate how burdened they are (never, rarely, sometimes, quite frequently, or nearly always). A total score can be calculated from the summing of each answer, with higher scores indicating higher levels of burden and stress because of the caring process [35,36].

Caregiver sleep quality will be examined with the *Pittsburgh Sleep Quality Index*, designed to evaluate the overall sleep quality for 1 month. It is a 19-item self-reported questionnaire with 7 subcategories: subjective sleep quality, sleep latency, sleep duration, habitual sleep efficiency, sleep disturbances, use of sleeping medication, and daytime dysfunction. This questionnaire was initially created to measure the sleeping quality in psychiatric populations but has been widely used for clinical and research purposes [37,38].

Caregivers’ satisfaction with the platform and usability of the system will be investigated via semistructured individual interviews conducted before and after the technology deployment, with the support of a previously designed interview guide that includes the following topics: (1) reason for participating in the project; (2) platform feedback on usability, usefulness, helpfulness, expectations, suitability, and improvements; (3) caregivers’ impressions of PwD perceptions of the CHESS platform; and (4) caregivers’ perceptions of home care technology in general. The interviews will be audio recorded for later transcription and analysis.

### Timing of Measurements

In both groups, PwD-caregiver dyads will be followed up for 12 months during which they will continue with their traditional care plan, but in addition, the intervention group will receive the CH package for their use at home during 6 months (months 3 to 9 of the yearly follow-up), thus enabling a baseline phase, intervention phase, and a postintervention phase to be delineated. During the initial assessment (month 0), PwDs’ past medical history and PwDs’ and informal caregivers’ social history and demographic data will be obtained. Further comprehensive assessments related to the caregivers’ and PwDs’ emotional and physical well-being will be performed at the initial assessment and at 3, 6, 9, and 12 months using the international and standardized validated questionnaires explained above. They will be completed electronically on the researchers’ administrators interface of the health platform by the caregiver and the patient, with the help of the researchers (see Table 1 for the comprehensive list of questionnaires and their timing during the 12 months follow-up, and see Multimedia Appendix 8 for a screenshot of the administrators’ interface view of the questionnaires). Respondent burden is a key consideration, and so in this way, all in-depth questionnaires are reserved for quarterly face-to-face interviews with the researchers, whereas daily and weekly questionnaires modules with which the caregiver interacts independently through the user interface are kept short and simple.

**Table 1:** Quarterly assessments and timing with the caregiver and person with dementia during the yearly follow-up.

Participant	Month 0	Month 3	Month 6	Month 9	Month 12
PwD^a^	Past medical history, vital signs; DEMQOL^b^, HADS^c^, MMSE^d^, QTUG^e^; Frailty (SHARE-FI^f^), DAD^g^, DEMQOL, proxy, NPI-Q^h^	DEMQOL, HADS, MMSE, QTUG, Frailty (SHARE-FI), DAD, DEMQOL proxy	DEMQOL, HADS, MMSE, QTUG, Frailty (SHARE-FI), DAD, DEMQOL proxy, NPI-Q	DEMQOL, HADS, MMSE, QTUG, Frailty (SHARE-FI), DAD, DEMQOL proxy	DEMQOL, HADS, MMSE, QTUG, Frailty (SHARE-FI), DAD, DEMQOL proxy, NPI-Q
Caregiver	EQ-5D-5L^i^, HADS, IPAQ^j^, NVS^k^, PSQI^l^, ZBI^m^	EQ-5D-5L, HADS, NVS, PSQI, ZBI	EQ-5D-5L, HADS, IPAQ, NVS, PSQI, ZBI	EQ-5D-5L, HADS, NVS, PSQI, ZBI	EQ-5D-5L, HADS, IPAQ, NVS, PSQI, ZBI

^a^PwD: person with dementia.

^b^DEMQOL: Dementia Quality of Life.

^c^HADS: Hospital Anxiety and Depression Scale.

^d^MMSE: Mini-Mental State Examination

^e^QTUG: Quantitative Timed Up and Go.

^f^SHARE-FI: Survey of Health, Ageing, and Retirement in Europe Frailty Instrument.

^g^DAD: Disability Assessment for Dementia.

^h^NPI-Q: Neuropsychiatric Inventory Questionnaire.

^i^EQ-5D-5L:EuroQol group-5 dimension- 5 level questionnaire.

^j^IPAQ: International Physical Activity Questionnaire.

^k^NVS: Newest Vital Signs.

^l^PSQI: Pittsburgh Sleep Quality Index.

^m^ZBI: Zarit Burden Interview.

### Initial Users’ Evaluation

A first model of the CH platform was developed over 12 months. Once the first stage of development of the platform was completed, we decided to conduct various levels of user testing with researchers and clinicians from our group to detect any possible platform failures or bugs, to verify that it worked according to our expectations, and to redefine the user interface based on their findings. During February 2017, 5 researchers and clinicians used the technology for 1 week, completing the tasks as the caregiver would and, also, wearing the activity tracker 24/7 as the PwD would. They were encouraged to think and to use the platform as a caregiver would and were provided with a list of the tasks to be completed during the week, that is, to measure their weight and to measure their BP at least once, to have a look at the information section (to watch some videos and to access some of the websites’ links provided), to write down minimum 1 entry in the journal, and to have a daily look at their activity and sleeping, so as to ensure that the devices were accurately syncing and being presented accurately in the measurements section. A log was created specifically for them to record any potential issues that appeared during the platform and device use, with the idea of solving them before the deployment phase. The platform seemed to work according to our scheme, and devices were syncing appropriately. Data were showed in the measurements section, and videos and links from the resources section were working properly. Researchers feedback was used to make some small changes in the platform’s look to give it a clearer and cleaner aspect and make it easier to use.

### Data Analysis

Quantitative data from the participants’ quarterly assessments and daily monitoring data will be exported from the platform in the form of Excel documents. Descriptive statistics will be used to summarize the initial demographic data from our sample, which are obtained from the medical notes and the initial assessment examination (month 0), and for describing the findings of the questionnaires at each assessment point (months 3-12). Exploratory factor analysis will be conducted on the measured variables to identify the main constructs in our sample and how they correlate and interact with each other.

The main data collection points will be at 2 stages: months 0 and 12, as they will give us insight into the progress of the disease and the quality of life of the PwD and caregivers over 1 year, whereas interim data points will be available at 3, 6, and 9 months. Thus, the primary analysis of these repeated measures within each individual will be assessed using linear mixed models (LMMs). LMMs are suitable for correlated data such as these, and random intercepts and slopes can be allowed for individuals and subgroups. Change over time will be assessed, and differences between subgroups and interactions between covariates will be explored.

We also want to investigate changes in the scores between each assessment point (at 0, 3 6, 9, and 12 months). For this, analysis of variance or the equivalent nonparametric tests will be used. Regarding the assessments at months 3 to 9, these are the months when the technology is deployed at their homes and in which we can assess any potential impact derived from its use between the intervention and the usual care groups. This group comparison will be estimated by comparing the change in the volunteers’ scores between those 2 assessments (month 3 and month 9) using *t* tests or the correspondent nonparametric tests when appropriate.

For this, a sample size calculation was derived using a rule of thumb for 20 observations for each dependent variable, so a sample size of 60 PwD-caregiver dyads is proposed. For the comparison of outcomes between the intervention and the usual care groups, caregiver burden and quality of life will be the primary outcome measure assessed at enrollment and at 6 and 12 months. Acknowledging that the PwD can deteriorate over time with a decline in their cognitive and functional status, we will use scores adjusted for baseline patient dependence and neuropsychiatric symptoms (analysis of covariance, ANCOVA model). We hypothesis a moderate between-groups effect size (*d*=0.5), so with 80% power and alpha=.05, a sample of 64 individuals in the intervention group and 64 in the usual care group is required for the *t* test. Adjusting for ANCOVA by sqrt (1-p2), where the p is the conservative correlation of 0.5 between repeated measures [39], results in a sample of 55 in each group. To account for dropout, 60 PwD-caregiver dyads will need to be recruited for each group. SPSS software (IBM Corp Version 24.0) will be used for this statistical analysis.

Analysis of the qualitative data obtained from the transcribed individual semistructured interviews will be analyzed using content analysis [40]. A guiding codebook will be used for this analysis, which will be created based on the topics examined in the interviews outlined above. The emerged categories will be organized, analyzed, and compared for generating themes. NVivo software version 12 for Macintosh (QSR International) will be used to organize the thematic analysis.

## Results

This 3-year funded study (2016-2019) is currently in its implementation phase. It is expected to finish by December 2019, and preliminary data results are expected to be submitted for publication by mid-2019. We believe that CH care approach can potentially change our current care model, moving from a previous passive and reactive approach to a more proactive and preventive model, utilizing self-management–based strategies, and enhancing caregivers’ involvement in the management of PwD’s health care in the home.

## Discussion

### Principal Findings

With this mixed methods study, we are addressing 2 spheres in the dementia informal home care. One is the invaluable quantitative health data collected from the PwD daily monitoring and the fact that, although the informal caregivers play the main role in PwD home care, there are many inconsistencies regarding the support provided to them. On the other sphere, the literature suggests that despite current trends in research claiming a user-centered design for increasing adoption and use [41], PwDs are not sufficiently involved in the design and development of CH solutions for dementia home care. Therefore, PwDs’ and their informal caregivers’ needs and perspectives are not considered and integrated into the product designed, so they are not customized for them [42,43]. Consequently, because of the created necessity of understanding the needs and attitudes of PwD and their informal caregivers regarding CH technology, we have included a qualitative approach to address these deficits outlined here. We hope that our combined results from both domains, qualitative and quantitative, will bring new perspectives on how to design and conduct CH research with PwDs and their informal caregivers.

### Conclusions

CH is emerging as a new approach to potentially change our current care model, moving from a previous passive and reactive approach to a more proactive and preventive model, utilizing self-management–based strategies, and enhancing caregivers’ involvement in the management of PwD’s health care in the home. In accordance with the *Chronic Care Model* [44], we foresee that our platform will provide knowledge and promote autonomy in the caregivers, which may empower them into greater control of the PwD care and, with it, improve the quality of life and well-being for the person they are caring for and themselves through a physical and cognitive decline predictive model. We also believe that facilitating information sharing between the health care professionals and the informal caregivers may enable a stronger relationship between all the stakeholders, facilitate a more coordinated care plan, and increase feelings of empowerment in the informal caregivers.

## Appendix

Multimedia Appendix 1 Applied Research in Connected Health, University College Dublin pilot project devices.

Multimedia Appendix 2 Devices used for the Connected HEalth Sustaining home Stay in Dementia project.

Multimedia Appendix 3 Caregivers’ platform main screen.

Multimedia Appendix 4 Information section.

Multimedia Appendix 5 Questionnaires section.

Multimedia Appendix 6 Profile section.

Multimedia Appendix 7 Measurements section.

Multimedia Appendix 8 Researchers’ assessments interface.

## Abbreviations

ADL: activity of daily living
ALADDIN: Assisted living of Dementia elDerly Individuals
ANCOVA: analysis of covariance
BP: blood pressure
CH: Connected Health
CHESS: Connected HEalth Sustaining home Stay in Dementia
EQ-5D-5L: Mini-Mental State Examination.
EQ-VAS: EuroQol group Visual Analogue Scale
HADS: Hospital Anxiety and Depression Scale
LMM: linear mixed model
NPI-Q: Neuropsychiatric Inventory Questionnaire
PwD: person with dementia
QTUG: Quantitative Timed Up and Go
UCD: University College Dublin
WHO: World Health Organization
