# Supplementary figures and images for: Development of a Caregivers’ Support Platform (Connected Health Sustaining Home Stay in Dementia): Protocol for a Longitudinal Observational Mixed Methods Study
Source: JMIR Res Protoc. 2019 Aug 28;8(8):e13280. doi: 10.2196/13280 (PMC6786855; doi:10.2196/13280)

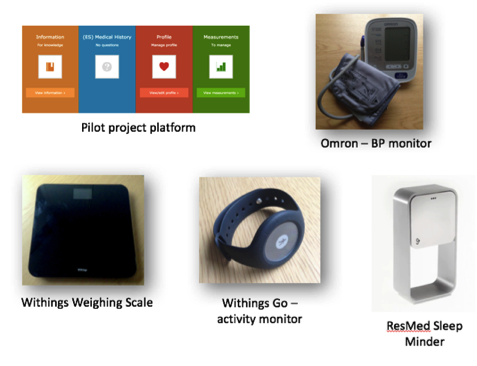

Supplement: Multimedia Appendix 1 [file resprot_v8i8e13280_app1.png]

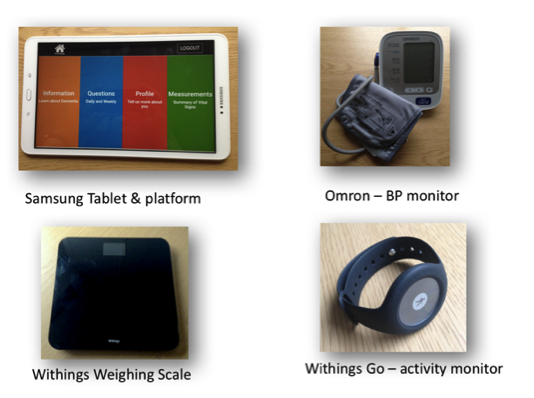

Supplement: Multimedia Appendix 2 [file resprot_v8i8e13280_app2.png]

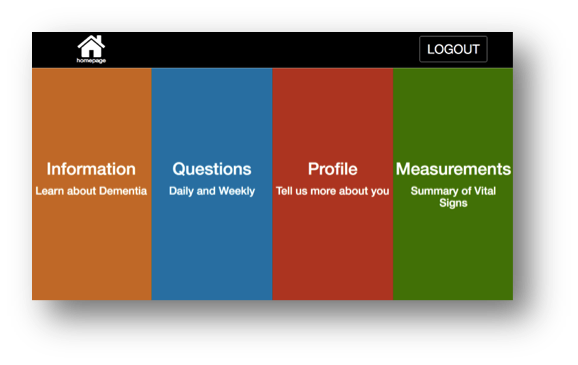

Supplement: Multimedia Appendix 3 [file resprot_v8i8e13280_app3.png]

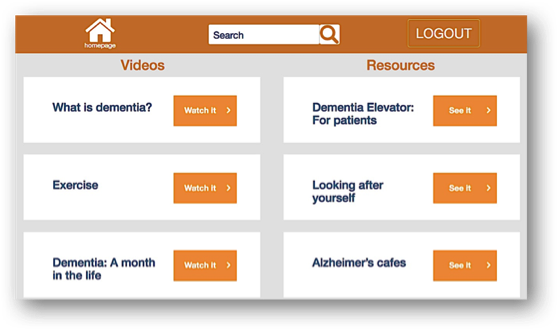

Supplement: Multimedia Appendix 4 [file resprot_v8i8e13280_app4.png]

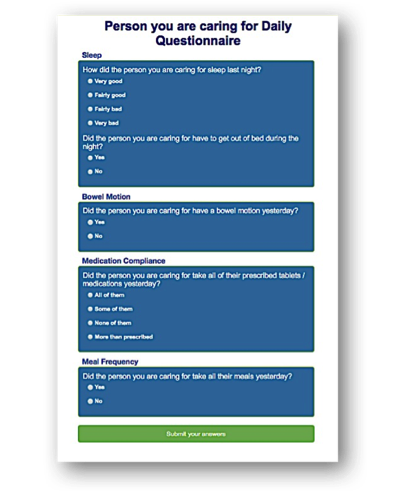

Supplement: Multimedia Appendix 5 [file resprot_v8i8e13280_app5.png]

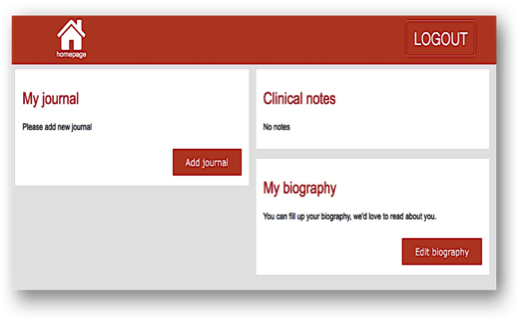

Supplement: Multimedia Appendix 6 [file resprot_v8i8e13280_app6.png]

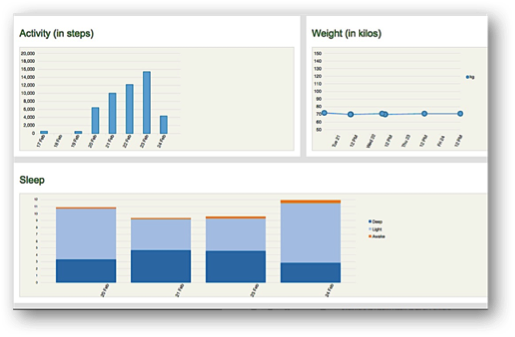

Supplement: Multimedia Appendix 7 [file resprot_v8i8e13280_app7.png]

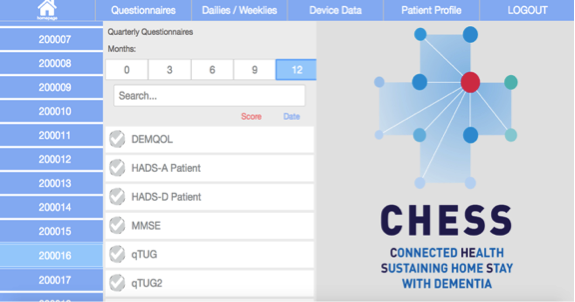

Supplement: Multimedia Appendix 8 [file resprot_v8i8e13280_app8.png]
